# Diversity and Antimicrobial Resistance Genotypes in Non-Typhoidal *Salmonella* Isolates from Poultry Farms in Uganda

**DOI:** 10.3390/ijerph15020324

**Published:** 2018-02-13

**Authors:** Terence Odoch, Camilla Sekse, Trine M. L’Abee-Lund, Helge Christoffer Høgberg Hansen, Clovice Kankya, Yngvild Wasteson

**Affiliations:** 1 Department of Bio-security, Ecosystems and Veterinary Public Health, College of Veterinary Medicine, Animal Resources and Biosecurity (COVAB), Makerere University, P.O. Box 7062, Kampala, Uganda; clokankya@yahoo.com; 2 Norwegian Veterinary Institute, 0106 Oslo, Norway; camilla.sekse@vetinst.no; 3 Department of Food Safety and Infection Biology, Faculty of Veterinary Medicine, Norwegian University of Life Sciences (NMBU), 0454 Oslo, Norway; trine.labee-lund@nmbu.no (T.M.L.-L.); helge.hansen@nmbu.no (H.C.H.H.); yngvild.wasteson@nmbu.no (Y.W.)

**Keywords:** antimicrobial resistance, genotypes, non-typhoidal *Salmonella*, poultry, genes, integrons, subtyping

## Abstract

Non-typhoidal *Salmonella* (NTS) are foodborne pathogens of global public health significance. The aim of this study was to subtype a collection of 85 NTS originating from poultry farms in Uganda, and to evaluate a subgroup of phenotypically resistant isolates for common antimicrobial resistance genes and associated integrons. All isolates were subtyped by pulsed-field gel electrophoresis (PFGE). Phenotypically resistant isolates (*n* = 54) were screened by PCR for the most relevant AMR genes corresponding to their phenotypic resistance pattern, and all 54 isolates were screened by PCR for the presence of integron class 1 and 2 encoding genes. These genes are known to commonly encode resistance to ampicillin, tetracycline, ciprofloxacin, trimethoprim, sulfonamide and chloramphenicol. PFGE revealed 15 pulsotypes representing 11 serotypes from 75 isolates, as 10 were non-typable. Thirty one (57.4%) of the 54 resistant isolates carried at least one of the seven genes (*bla*_TEM-1,_ *cmlA, tetA, qnrS,* *sul1,* *dhfrI,* *dhfrVII)* identified by PCR and six (11%) carried class 1 integrons. This study has shown that a diversity of NTS-clones are present in Ugandan poultry farm settings, while at the same time similar NTS-clones occur in different farms and areas. The presence of resistance genes to important antimicrobials used in human and veterinary medicine has been demonstrated, hence the need to strengthen strategies to combat antimicrobial resistance at all levels.

## 1. Introduction

*Salmonella enterica* subsp. *enterica* include serotypes that are global foodborne pathogens significantly affecting public health and economy [1,2,3]. In humans, salmonellosis is classified into typhoid and non-typhoidal salmonellosis. Most cases of non-typhoidal *Salmonella* (NTS) disease are associated with consumption of contaminated foods of animal origin, particularly poultry, meat and in some instances vegetables [4,5,6]. Globally, NTS is estimated to cause 93.8 million cases of gastroenteritis annually, of which 80 million cases are foodborne and causing 155,000 deaths [7]. Although African countries have low estimated cases of NTS gastroenteritis compared to other parts of the world, they have a much higher level of invasive non-enteric NTS infections [7,8]. NTS bacteraemia is an emerging opportunistic infection in individuals infected with HIV and is reported to be highly correlated with malaria, especially in children and elderly persons [9,10,11,12,13].

In poultry, transmission of NTS can occur by direct contacts with infected birds, consumption of contaminated feeds and water, and contact with environmental reservoirs [13]. Transmission can also occur through cross contamination anywhere along the production chain, and for specific serotypes, vertical transmission is also possible [14,15]. However, NTS infections in poultry is mainly asymptomatic [14], and may therefore not get the necessary attention with regard to prevention and control. The diversity of NTS circulating in poultry and livestock production environment in most developing countries is poorly understood, as very limited studies have been undertaken. Molecular typing is important for characterization of bacteria to establish genetic relatedness between isolates in order to elucidate the dynamics of the bacterial populations. Although whole genome sequencing is getting more established, pulsed-field gel electrophoresis (PFGE) technique is still considered an adequate molecular method suitable for subtyping of serotypes of *Salmonella*.

The increasing development of antimicrobial resistance (AMR) in NTS is complicating treatment of bacteraemia cases and results in poorer treatment outcomes. Even more worrying is the emergence of multidrug resistance (MDR) in NTS against commonly used antibiotics in human and animal treatment, which has become a serious public health challenge [15,16,17,18]. Resistance is increasing not only against first line antibiotics, but also against clinically important antimicrobial agents like fluoroquinolones and third generation cephalosporins [19]. Inappropriate use of antimicrobials in agriculture is known to be a key factor contributing to the development of AMR, and the influence of livestock environment in the development of MDR in NTS has been demonstrated [20]. Increased intensification of production in agriculture, use of antibiotics as feed additives, and prophylactic treatment are some of the practices that influence development of AMR [21,22]. MDR NTS can be transferred from the poultry reservoirs to humans through the food chain, but AMR can also be transferred from one bacterium to another through resistance genes associated with integrons and mobile genetic elements such as plasmids and transposons. Most studies on AMR in poultry are done in developed countries while in most developing countries, including Uganda, there are no surveillance and monitoring programs for important foodborne pathogens and AMR in primary production units. To date in Africa, only a few limited studies have documented AMR and corresponding genes in NTS isolated from humans, animal products, and poultry farms [23,24,25,26,27,28,29]. Therefore, data is scarce and the extent of NTS and AMR remains poorly known. As a result, development of appropriate mitigation measures and control efforts is compromised. The aim of this study was to characterize a collection of NTS isolates from poultry by using PFGE for molecular subtyping and to investigate the presence of integrons and acquired antimicrobial resistance genes from the phenotypically resistant isolates. The NTS were isolated from faecal samples collected from poultry farms in three districts (Wakiso, Lira, and Masaka) in Uganda between 2015 and 2016 [30].

## 2. Materials and Methods

### 2.1. The NTS isolate collection

The majority (75/85) of the NTS isolates used in this study were from a previous study by Odoch et al. [30]. The remaining 10 isolates originated from additional sampling. However, all 85 isolates were from fecal samples collected from poultry houses in three districts with high numbers of commercial poultry farms (Wakiso, Lira, and Masaka) in Uganda between 2015 and 2016, according to a sampling design and procedure described in Odoch et al [30]. A map of the study area is provided as Supplementary Materials Figure S1. NTS were isolated, identified, serotyped and tested for antimicrobial sensitivity according to standard methods as earlier described [30]: Culture and isolation of NTS were done according to ISO 6579:2002/Amd 1:2007, Annex D: Detection of *Salmonella* spp. in animal faeces and in environmental samples from the primary production [31]. Biochemical confirmatory tests were done by using the API-20E (BioMerieux, Marcy l’Etoile, France) identification system. All isolates were serotyped according to the Kauffman–White–Le–Minor technique at the Norwegian Veterinary Institute. Phenotypic susceptibility testing of 13 antimicrobials (gentamicin, sulonamide, trimethoprim-sulfamethoxazole, ciprofloxacin, cefotaxime, meropenem, chloramphenicol, ceftazidime, ampicillin, amoxicillin/clavulanic acid, trimethoprim, tetracycline, and enrofloxacin) was performed by the disc diffusion test. The metadata, serotype and phenotypic resistance of the isolates are presented in the Supplementary Materials (Table S1).

### 2.2. Pulsed-Field Gel Electrophoresis (PFGE) and Bionumerics Analysis

The PulseNet standardized protocol for PFGE for molecular subtyping of *Salmonella* (https://www.cdc.gov/pulsenet/pathogens/pfge.html) was used on all the 85 isolates. Overnight cultures were used to prepare DNA templates according to the PulseNet protocol. DNA was digested with the restriction enzyme *XbaI* and *Salmonella* Braenderup H9812 was used as a molecular size standard in all PFGE investigations. Electrophoresis was performed with the CHEF-DR III system (Bio-Rad Laboratories, Hercules, CA, USA) with the following set parameters: initial switch time 2.2 s, final switch time 63.8 s, voltage-6 V, time-19 h and temperature 14 °C. The gels were stained with ethidium bromide and the bands visualized under UV transillumination and captured by GelDoc EQ system with Quantity One® software (Version 4.2.1; Bio-Rad Laboratories, Hercules, CA, USA). PFGE banding patterns were compared using a combination of visual inspection and the BioNumerics software vers. 6.6.11 (Applied Maths, Ghent, Belgium). A dendrogram was generated using band-based dice similarity coefficient and the unweighted pair group method using a geometric average (UPGMA) with 1.2% position tolerance and 1.2% optimization. A cutoff of 97% similarity was used to define a PFGE pulsotype (PT).

### 2.3. Bacterial DNA Extraction

Total DNA for PCR were extracted using the boiled lysate method [32]. This was done by taking 200 µL of an overnight culture, mixing with 800 µL of sterile distilled water and boiling for 10 minutes. The resultant solution was centrifuged at 13,000 rpm for five minutes and the supernatant was used as a DNA template. This was kept at −20 °C for subsequent use.

### 2.4. Detection of Integrons and Antibiotic Resistance Genes

The isolates that were classified as resistant according to the results of the disc diffusion test (*n* = 54) were screened by PCR for the most relevant AMR genes corresponding to their phenotypic resistance pattern. In addition, all resistant isolates were screened by PCR for the presence of integron class 1 and 2 encoding genes. The isolates tested were *S.* Newport (*n* = 18), *S.* Bolton (*n* = 8), *S.* Hadar (*n* = 6), *S.* Mbandaka (*n* = 4), *S.* Heidelberg (*n* = 8), *S.* Typhimurium (*n* = 2), and *S.* Zanzibar (*n* = 8) serotypes. The existence of class 1 integron was investigated by PCR for the detection of genes encoding the variable part between the 5’ conserved segment and the 3’ conserved segment of the variable region [33]. Presence of class 2 integron was investigated by detection of *hep74* and *hep51* genes using primers and following PCR conditions previously reported [33]. Presence of 22 AMR genes (Table 1) known to confer resistance to six commonly used classes of antimicrobials (β-lactams, tetracyclines, phenicols, fluoroquinolones, trimethoprim, and sulfonamides) were investigated by PCR. The primer sets used for detection of integrons and AMR genes are shown in Table 1. Ampicillin resistant isolates (*n* = 4) were screened for four β-lactamase resistance encoding genes, and ciprofloxacin resistant isolates (*n* = 40) were screened for four fluoroquinolone plasmid mediated quinolone resistance (PMQR) determinant genes. Chloramphenicol resistant isolates (n=4) were screened for four phenicol resistance genes, tetracycline resistant isolates (n=12) were screened for three genes. Sulfonamide resistant isolates (*n* = 21) were screened for two genes and six trimethoprim resistant isolates were screened for five trimethoprim resistance genes. These genes were selected because they are the most frequently detected genes associated with the corresponding phenotypes of the NTS isolates [34]. All the integron PCR products were purified and sequenced (GATC Biotech, Cologne, Germany) and the sequence results were analysed using BLAST and compared to GenBank database (http://blast.ncbi.nlm.nih.gov/blast.cgi). Similarly, one PCR product from each of the AMR PCRs was sequenced to confirm the PCR results. Negative controls were included in all PCR analyses.

The β-lactamase encoding genes (*blaPSE-1, blaCMY-2, bla*_TEM-1_, *blaOxA*) encode production of β-lactamase enzyme that breaks the β-lactam antibiotic ring open and deactivates the molecule’s antibacterial properties. The plasmid mediated quinolone resistance genes (*qnrA, qnrB, qnrC, qnrS*) encode pentapeptide repeat proteins that bind to and protects DNA gyrase and topoisomerases IV from the inhibition of quinolones. The phenicol resistance genes, (*cat1, cat2*) encode chloramphenicol acetyltransferase enzyme that inactivates chloramphenicol, chloramphenicol resistance gene, *cmlA* and florfenicol resistance gene *floR*, encode efflux pump proteins. Sulfonamide resistance genes *sul1* and *sul2* encode insensitive sulfonamide-resistant dihydropteroate synthase which cannot be inhibited by sulfonamide. Tetracycline resistance genes (*tetA, tetB, tetG)* encode membrane associated efflux pump proteins that export tetracycline from the cell and reduces drug concentration and thereby protecting ribosomes. Trimethoprim resistance genes *(dhfrI, dhfrV, dhfrVII, dhfrIX, dhfrXIII*) encode a drug-insensitive dihydrofolate reductase which cannot be inhibited by trimethoprim.

## 3. Results

### 3.1. Pulsed-Field Gel Electrophoresis Typing

**Figure 1:**
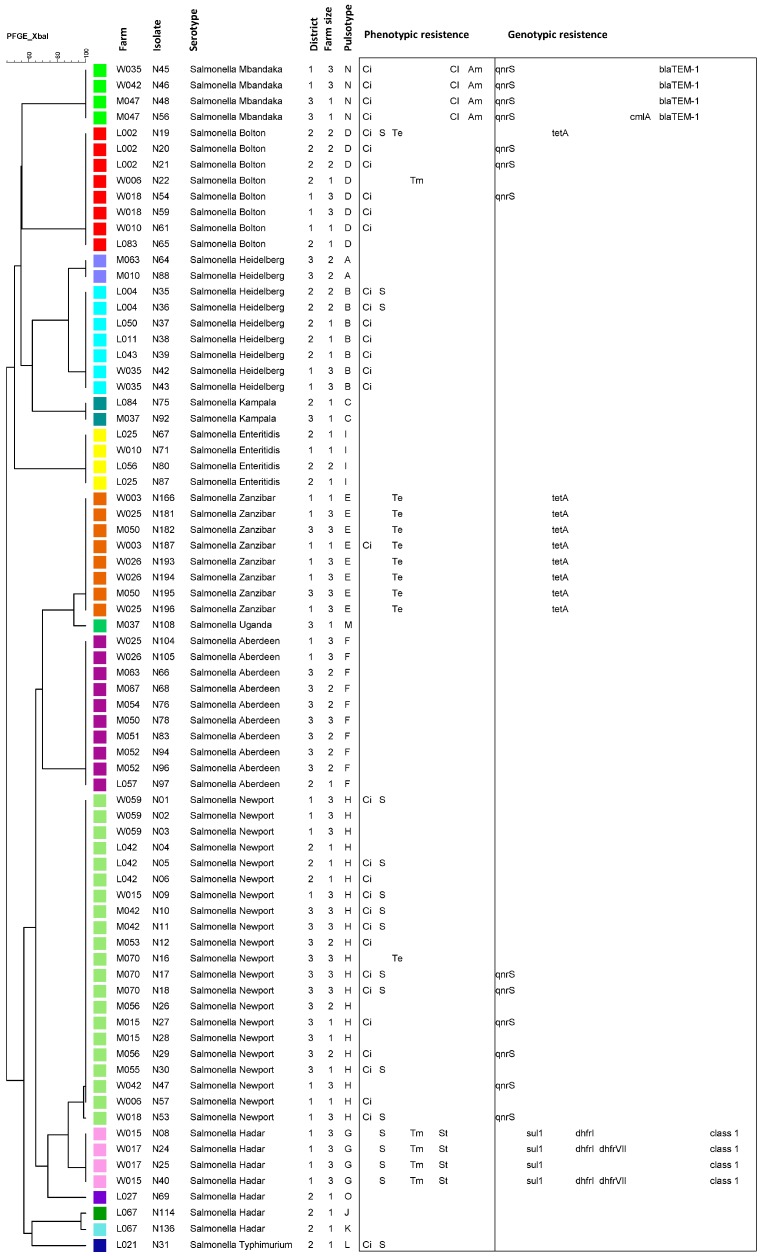
Dendrogram based on Pulsed-Field gel electrophoresis (PFGE) patterns of 75 non-typhoidal Salmonella from poultry from Uganda. A cutoff level of 97% similarity defines a PFGE profile. For each isolate the isolate number, PFGE profile, serotype, farm, size of farm, district, phenotypic resistance (Ci; ciprofloxacin, S; sulphonamide, Te; tetracycline, Tm; trimethoprim, St; sulphamethoxazole_trimethoprim, Cl; chloramphenicol, Am; ampicillin) and identified genotypic resistance genes (*qnrS, sul1, tetA, dhfrI, dhfrVII, cmlA*, *bla*_TEM-1_*, integrons, dfrA15, aadA1*) have been included.

A total of 75 Salmonella isolates were typable, and 15 PTs were identified (Figure 1) and the PFGE banding pattern of all isolates were included in a dendrogram as the Supplementary Materials (Figure S2). The 10 nontypable (NT) isolates belonged to different serotypes; Salmonella Bolton (*n* = 1), *S.* Newport (*n* = 3), *S.* Typhimurium (*n* = 1), *S.* Hadar (*n* = 4), and *S.* Heidelberg (*n* = 1). For the majority of the typable isolates, there was a complete association between serotype and PT. The 21 typable *S.* Newport isolates all belonged to PT (H), but were isolated from several farms in all districts (Figure 1). Ciprofloxacin resistant isolates were the majority and most diverse in terms of serotypes, pulsotypes and geographic distribution. Four *S.* Mbandaka isolates were characterized by the same PT (N) and phenotypic resistance pattern, but were isolated from three different farms in two districts. A similar distribution pattern was also observed for 10 *S.* Aberdeen isolates of PT (F); these were isolated from nine different farms from all districts. However, the isolates were fully sensitive in the disc diffusion test. The exceptions from the serotype-PT associations were *S.* Hadar and *S.* Heidelberg. A total of seven *S.* Hadar isolates were typable. Four of them with identical PT originated from the same district, but from two farms, and had same phenotypic resistance towards three antimicrobials. The other three *S.* Hadar isolates had three different PTs, however, two of these isolates were similar with only one band difference (Figure S2). The typable *S.* Heidelberg isolates consisted of two different PTs; one PT (A) with two isolates from the same district and one PT (B) with seven isolates from the other two districts. The isolates in PT (A) were fully susceptible in the disc diffusion test, while all in PT (B) expressed ciprofloxacin resistance and two also expressed sulfonamide resistance.

### 3.2. Detection of Integrons and Antibiotic Resistance Genes

Genes encoding class 1 integrons were only detected in six *S.* Hadar isolates, four belonging to PT (G) and two nontypable. The integrons were similar in size, with approximately 1700 bp. All the *S.* Hadar isolates that carried integrons originated from four farms in one district, Wakiso. Genes encoding class 2 integrons were not detected in any of the isolates. Sequencing of the six integron PCR products revealed the presence of *aadA1* and *dfrA15* genes that confer resistance to streptomycin/spectinomycin and trimethoprim, respectively. 

AMR genes were detected in 31 (57.4%) of the 54 phenotypically resistant. Only seven genes *(bla*_TEM-1_*, cmlA, qnrS, tetA, sul1, dhfrI, dhfrVII)* of the 22 AMR genes were detected among the selected phenotypically resistant isolates. These genes are known to confer resistance to six categories of antimicrobials (β-lactams, chloramphenicol, fluoroquinolones, tetracyclines, sulfonamides, and trimethoprim).

All four ampicillin and chloramphenicol resistant *S.* Mbandaka strains harbored the *bla*_TEM_ gene that confers resistance to β-lactams, but only one of them was harboring the chloramphenicol resistance gene *cmlA.* The PMQR gene *qnrS* was detected in 16 (18.8%) out of the total 85 isolates. Forty of these displayed ciprofloxacin resistance, of which 16 (40%) carried *qnrS*. All 13 tetracycline resistant isolates were positive for the *tetA* gene. The sulfonamide resistant gene *sul1,* was the only one identified in six of the 21 sulfonamide resistant isolates (*sul2* was not detected). Out of the six trimethoprim resistant *S.* Hadar strains, four were resistant to sulfonamide/trimethoprim and they all harbored the *dhfr*1 gene (Table S1). Three of the six harbored both *dhfr1* and *dhfrVII* (Figure 1).

## 4. Discussion

The diversity of NTS circulating in poultry in most developing countries is poorly understood, as few studies have been undertaken [44,45,46]. In this study, 15 PTs from 11 different serotypes of NTS isolates were identified, with most of the identified serotypes having only one PT implying they are clonally related. The PFGE dendrogram combined with the geographical origin of the isolates indicate that many related clones are circulating in geographically diverse areas. For example *S*. Newport, the most prevalent serotype of all, belonged to the same PT and was isolated from all the districts. This situation is not surprising considering the uncontrolled movement of poultry and poultry products in Uganda. In addition, most commercial farms share sources of chicks, feeds, feed ingredients, and live bird markets and these are all potential common sources of NTS contamination. A similar situation has been reported in Senegal [46]. Because NTS is known to persist in the environments for months [47,48], they can easily be spread over large geographical areas. Some of the NTS serotypes represented in this study have caused foodborne illnesses and outbreaks globally [49]. There were isolates with similar PTs that varied with regard to their content of resistance genes, the AMR genes tested for are acquired genes, and not through mutations in chromosomally encoded genes, therefore the genes might be spread among isolates due to their location on plasmids, transposons and integrons. Integration of these elements does not necessarily result in changes in PT.

Through this study, the occurrence of AMR genes among a diversity of NTS isolates from poultry farms in the study districts have been unveiled. The isolates were screened for the genes conferring resistance to the antibiotics to which the isolate revealed a resistance phenotype. The genes detected confer resistance to some of the most important antimicrobials used for treatment of bacterial infections in humans and animals [50]. However, among the 22 AMR genes that are commonly occurring within the *Enterobacteriaceae* family, only seven genes were identified. Discordance was seen where observed phenotypic AMR was not reflected by the detection of corresponding AMR genes. For example, neither *sul1* nor *sul2* genes were detected in the nine phenotypically sulfonamide resistant *S*. Newport isolates. This discordance could be due to presence of other and more unusual resistance mechanisms encoded by genes not included in this study.

Previous investigations on the occurrence of integrons in NTS isolates from animal sources have yielded varying results [51,52,53]. Class 1 integrons are known for their roles in the dissemination of AMR, especially in the carrying of multiple AMR genes. In this study, integrons were identified in six *S.* Hadar isolates and all of them were identified with *aadA1* and *dfrA15* genes that confer resistance to streptomycin/spectinomycin and trimethoprim, respectively. It is in agreement with studies and reports that most of these genes are found in gene cassettes located within class 1 and 2 integrons [41,51]. In addition, PCR identified four of these *S.* Hadar isolates with *dhfrI* genes with three of the four carrying both *dhfrI* genes and *dhfrVII* genes. More than 30 gene variants encoding dihydrofolate reductase have been identified [38] and *dfrA* are the most commonly genes identified from NTS.

Class I integrons are always associated with *sul1* genes. In this study, *sul1* gene was the only sulfonamide resistance gene identified in six of the 21 phenotypically sulfonamide resistant isolates. Previous studies have reported that in NTS, *sul1* is more common than *sul2* and *sul3* and these genes encode the dihydropteroate synthase [54]. As reported earlier, increase in resistance to sulfonamides/trimethoprim in Uganda has serious public health implications as it is the main drug used to control opportunistic infections in HIV/AIDS patients [30].

The PMQR gene *qnrS* was the only PMQR gene detected from the NTS isolates that were phenotypically resistant to ciprofloxacin. This finding is in agreement with some similar studies undertaken previously [55,56,57]. It may, however, be noted that the detection of the *qnrS* genes was restricted to the serotypes *S*. Newport, *S*. Bolton and *S*. Mbandaka, while they were not detected in *S*. Zanzibar, *S*. Typhimurium, *S*. Heidelberg. PMQR genes are rapidly spreading globally, although their presence only mediate low levels of fluoroquinolone resistance, they can interact with genomic determinants to increase the minimum inhibitory concentrations of fluoroquinolones of the PMQR harboring bacteria [58]. Ciprofloxacin is an important fluoroquinolone used in Uganda and other countries for treatment of salmonellosis and other bacteraemic infections. It is often used as a last resort antimicrobial in the treatment of blood stream infections in children and is classified by World Health Organization (WHO) as critically important [50]. In the current study areas, a potential risk exits that ciprofloxacin resistance genes could get transferred to humans through contact with poultry, and consequently complicate the use of ciprofloxacin. The high occurrence of *qnrS* in NTS from poultry needs to be explored further to determine whether it could be associated with use of enrofloxacin in poultry. Enrofloxacin, also a fluoroquinolone, is sometimes used prophylactically and metaphylactically in combination with other drugs in some commercial poultry farms in Uganda [30]. As all fluoroquinolones have the same mechanism of inhibition of the topoisomerase genes, resistance to any one of them will confer resistance to all others. High presence of the plasmid-mediated quinolone resistance gene *qnrS* therefore shows the potential of horizontal transfer of resistance genes [59].

In this study, all the tetracycline resistant isolates carried *tetA* genes, they were all negative for *tetB* and *tetG* genes. This result is similar to what has been reported in previous studies undertaken in Thailand, Australia, Germany, Morocco, and Egypt [18,60,61,62,63]. However, the results is also in contrast to another study in Egypt [64]. Many genes responsible for tetracycline resistance have been identified and described [65]. The occurrence of *tetA* gene is known to be widespread in NTS and is associated with non-conjugative transposons. These genes are associated with efflux pump mechanisms implying that these are the predominant mechanisms for tetracycline resistance in NTS in these areas. High presence of *tetA* genes is not surprising as tetracycline is an extensively used drug in human and veterinary medicine, mainly because it is cheap and readily available [66].

All four *S*. Mbandaka isolates that were resistant to chloramphenicol were negative for phenicol resistance encoding genes *floR, cat1, cat2*, and only one was positive for *cmlA* genes. This finding is consistent with an earlier study [67]. The chloramphenicol exporter gene *cmlA* has been previously found in plasmid-located class 1 integrons in *S.* Typhimurium. Use of chloramphenicol for animal treatment is banned in many countries, including Uganda, due to health hazards associated with the persistence of residues in foods [68]. These same isolates of *S*. Mbandaka were identified with *qnrS* gene and *bla*_TEM-1_ gene but were negative for all the other screened β-lactamase encoding genes( *bla*_PSE-1_, *bla*_CMY-2_, bla_OxA)_. The gene *bla*_TEM-1,_ is reported to be the most widely distributed of the β-lactamase genes worldwide [52] and is mainly known to be spread by plasmids. Not much information is available on the occurrence of beta-lactamase encoding genes in isolates from poultry in Uganda, but similar results have been reported in studies elsewhere [69,70,71]. Carriage of the *bla*_TEM-1_ gene is a threat to the potency of β-lactam antibiotics and in the case of Uganda, ampicillin is still widely used in human and veterinary medicine.

The interpretation of results from this study needs to be taken with a bit of caution, especially when looking at the bigger picture of the whole country. This study evaluated a limited number of resistance genes and only on phenotypically resistant isolates from a previous study [30], the sample size was quite small and samples were collected from only three districts that were purposively selected. However, as far as we are concerned, it is the first of its kind in Uganda and the data generated should make a significant contribution towards the national and international efforts to control antimicrobial resistance.

## 5. Conclusions

This study was a follow up of a previous study that determined prevalence, antimicrobial susceptibility and risk factors associated with NTS in Uganda [30]. The occurrence of AMR genes and integrons in *Salmonella enterica* isolates from Ugandan poultry has been unveiled, and through subtyping, the diversity of NTS isolates from three districts in Uganda has been explored.

The study has put into perspective the need to monitor use of antimicrobials and occurrence of AMR genes in farm ecosystems in developing countries, in order to institute measures to contain spread of AMR. Poultry keeping is predicted to continue growing in developing countries and in Uganda it will remain an important economic activity. However, as demonstrated, poultry farm environments remain a significant source of spread of AMR genes. Farmers have to be educated on the adoption of strict biosecurity measures, prudent use of antimicrobials and better management practices. More investigations need to be undertaken to further enhance understanding of the driving forces in farm ecosystems for the development of AMR in important foodborne pathogens like *Salmonella.* This study underscores the need for using the One Health approach to generate data on AMR in *Salmonella* organisms originating from humans, animals, and environmental samples.

## Figures and Tables

**Table 1:** PCR primers used for amplification of genes encoding integrons and antimicrobial resistance in non-typhoidal *Salmonella* isolates.

Target Category	Target Gene	Primer Sequence	Amplicon Size (bp)	Annealing Temp (°C)	Reference
Integron	Class 1 integron		Variable size	55	[33]
	*5’-CS*	GGCATCCAAGCAGCAAG			
	*3’-CS*	AAGCAGACTTGACCTGA			
	Class 2 integron		491	55	[33]
	*hep74*	CGGGATCCCGGACGGCATGCACGATTTGTA			
	*hep51*	GATGCCATCGCAAGTACGAG			
Resistance to ampicillin by detection of four β-lactamase genes	*bla*_PSE-1_	CGCTTCCCGTTAACAAGTAC	419	57	[35]
		CTGGTTCATTTCAGATAGCG			
	*bla*_CMY-2_	TGGCCAGAACTGACAGGCAAA	462	64	[36]
		TTTCTCCTGAACGTGGCTGGC			
	*bla*_TEM-1_	AGGAAGAGTATGATTCAACA	535	55	[37]
		CTCGTCGTTTGGTATGGC			
	*bla*_OxA_	ACCAGATTCAACTTTCAA	590	55	[38]
		TCTTGGCTTTTATGCTTG			
Resistance to ciprofloxacin by detection of four fluoroquinolone plasmid mediated quinolone resistance genes	*qnrA*	AGAGGATTTCTCACGCCAGG	580	54	[39]
		TGCCAGGCACAGATCTTGAC			
	*qnrB*	GATCGTGAAAGCCAGAAAGG	476	53	[40]
		ATGAGCAACGATGCCTGGTA			
	*qnrC*	GGGTTGTACATTTATTGAATCG	307	53	[40]
		CACCTACCCATTTATTTTCA			
	*qnrS*	GCAAGTTCATTGAACAGGGT	428	54	[39]
		TCTAAACCGTCGAGTTCGGCG			
Resistance to chloramphenicol by detection of four phenicol resistance genes	*floR*	AACCCGCCCTCTGGATCAAGTCAA	548	60	[41]
		CAAATCACGGGCCACGCTGTATC			
	*cat1*	CTTGTCGCCTTGCGTATAAT	508	55	[42]
		ATCCCAATGGCATCGTAAAG			
	*cat2*	AACGGCATGATGAACCTGAA	547	55	[42]
		ATCCCAATGGCATCGTAAAG			
	*cmlA*	CGCCACGGTGTTGTTGTTAT	394	55	[42]
		GCGACCTGCGTAAATGTCAC			
Resistance to sulfonamide by detection of two dihydropteroate reductase genes	*sul1*	GCG CGG CGT GGG CTA CCT	350	65	[43]
		GATTTCCGCGACACCGAGACAA			
	*sul2*	CGG CAT CGT CAA CAT AACC	720	52	[43]
		GTG TGC GGA TGA AGT CAG			
Resistance to tetracycline by detection of three efflux pump genes	*tetA*	GCTACATCCTGCTTGCCTTC	210	55	[35]
		CATAGATCGCCGTGAAGAGG			
	*tetB*	TTGGTTAGGGGCAAGTTTTG	659	55	[35]
		GTAATGGGCCAATAACACCG			
	*tetG*	CAG CTTTCG GATTCT TACGG	844	55	[35]
		GAT TGGTGA GGCTCG TTAGC			
Resistance to trimethoprim by detection of five dihydrofolate reductase genes	*dhfrI*	AAGAATGGAGTTATCGGGAATG	391	50	[37]
		GGGTAAAAACTGGCCTAAAATTG			
	*dhfrV*	CTGCAAAAGCGAAAAACGG	432	50	[37]
		AGCAATAGTTAATGTTTGAGCTAAAG			
	*dhfrVII*	GGTAATGGCCCTGATATCCC	265	50	[37]
		TGTAGATTTGACCGCCACC			
	*dhfrIX*	TCTAAACATGATTGTCGCTGTC	452	50	[37]
		TTGTTTTCAGTAATGGTCGGG			
	*dhfrXIII*	CAGGTGAGCAGAAGATTTTT	294	50	[37]
		CCTCAAAGGTTTGATGTACC			

## Supplementary Materials

The following are available online at http://www.mdpi.com/1660-4601/15/2/324/s1, Figure S1: A map of the study areas, Figure S2: A PFGE dendrogram of all typable isolates including the PFGE banding pattern, Table S1: List of all Salmonella isolates with metadata.
