# Supplementary material for: Diversity and Antimicrobial Resistance Genotypes in Non-Typhoidal Salmonella Isolates from Poultry Farms in Uganda
Source: Int J Environ Res Public Health. 2018 Feb 13;15(2):324. doi: 10.3390/ijerph15020324 (PMC5858393; doi:10.3390/ijerph15020324)
Supplement: Supplementary file 1 [file ijerph-15-00324-s001.zip › ijerph-257722-sup.Table S1.pdf]

Table S1: Non-typhoidal *Salmonella* isolates from Uganda 2017 showing phenotypic and genotypic antimicrobial resistance profiles for selected antimicrobials

Sample_id	Farm_id	Farm Size	District	Serotype	Phen.Res	Res.genes	Integrons
N01	W059	3	Wakiso	S. Newport	CIP,SUL		
N02	W059	3	Wakiso	S. Newport			
N03	W059	3	Wakiso	S. Newport			
N04	L042	1	Lira	S. Newport			
N05	L042	1	Lira	S. Newport	CIP,SUL		
N06	L042	1	Lira	S. Newport	CIP		
N08	W015	3	Wakiso	S. Hadar	SUL,TRIM,SXT	*sul1,dhfr1,*	*class1(dfrA15,aadA1)*
N09	W015	3	Wakiso	S. Newport	CIP,SUL		
N10	M042	3	Masaka	S. Newport	CIP,SUL		
N104	W025	3	Wakiso	S. Aberdeen			
N105	W026	3	Wakiso	S. Aberdeen			
N108	M037	1	Lira	S. Uganda			
N11	M042	3	Masaka	S. Newport	CIP,SUL		
N114	L067	1	Lira	S. Hadar			
N12	M053	2	Masaka	S. Newport	CIP		
N128	L067	1	Lira	S. Hadar			
N13	W019	3	Wakiso	S. Newport	CIP,SUL,TET,ENR,SXT	*qnrS,tetA*	
N136	L067	1	Lira	S. Hadar			
N14	W019	3	Wakiso	S. Newport	CIP,TET,ENR	*qnrS,tetA*	
N15	W019	3	Wakiso	S. Newport	CIP,TET,ENR	*qnrS,tetA*	
N16	M070	3	Masaka	S. Newport	TET		
N166	W003	1	Wakiso	S.Zanzibar	TET	*tetA*	
N17	M070	3	Masaka	S. Newport	CIP,SUL	*qnrS*	
N18	M070	3	Masaka	S. Newport	CIP,SUL	*qnrS*	
N181	W025	3	Wakiso	S.Zanzibar	TET	*tetA*	

N182	M050	3	Masaka	S.Zanzibar	TET	*tetA*	
N187	W003	1	Wakiso	S.Zanzibar	CIP,TET	*tetA*	
N19	L002	2	Lira	S. Bolton	CIP,SUL,TET	*sul1,tetA*	
N193	W026	3	Wakiso	S.Zanzibar	TET	*tetA*	
N194	W026	3	Wakiso	S.Zanzibar	TET	*tetA*	
N195	M050	3	Masaka	S.Zanzibar	TET	*tetA*	
N196	W025	3	Wakiso	S.Zanzibar	TET	*tetA*	
N20	L002	2	Lira	S. Bolton	CIP	*qnrS*	
N21	L002	2	Lira	S. Bolton	CIP	*qnrS*	
N22	W006	1	Lira	S. Bolton	TRIM		
N23	W017	3	Wakiso	S. Hadar	SUL,TRIM,SXT	*sul1,dhfr1*	*class1(dfrA15,aadA1)*
N24	W017	3	Wakiso	S. Hadar	SUL,TRIM,SXT	*sul1,dhfr1,dhfrVII*	*class1(dfrA15,aadA1)*
N25	W017	3	Wakiso	S. Hadar	SUL	*sul1*	
N26	M056	2	Masaka	S. Newport			
N27	M015	1	Masaka	S. Newport	CIP	*qnrS*	
N28	M015	1	Masaka	S. Newport			
N29	M056	2	Masaka	S. Newport	CIP	*qnrS*	
N30	M055	1	Masaka	S. Newport	CIP,SUL		
N31	L021	1	Lira	S. Typhimurium	CIP,SUL	*sul1*	
N32	L021	1	Lira	S. Typhimurium	SUL		
N35	L004	2	Lira	S. Heidlberg	CIP,SUL		
N36	L004	2	Lira	S. Heidlberg	CIP,SUL		
N37	L050	1	Lira	S. Heidlberg	CIP		
N38	L011	1	Lira	S. Heidlberg	CIP		
N39	L043	1	Lira	S. Heidlberg	CIP		
N40	W015	3	Wakiso	S. Hadar	SUL,TRIM,SXT	*sul1,dhfr1,dhfrVII*	*class1(dfrA15,aadA1)*
N41	W015	3	Wakiso	S. Hadar	SUL,TRIM,SXT	*sul1,dhfrVII*	*class1(dfrA15,aadA1)*

N42	W035	3	Wakiso	S. Heidlberg	CIP		
N43	W035	3	Wakiso	S. Heidlberg	CIP		
N44	W035	3	Wakiso	S. Heidlberg	CIP,ENR		
N45	W035	3	Wakiso	S. Mbandaka	CIP,CLR,AMP	*qnrS,blaTEM-1*	
N46	W042	3	Wakiso	S. Mbandaka	CIP,CLR,AMP	*qnrS,blaTEM-1*	
N47	W042	3	Wakiso	S. Newport			
N48	M047	1	Masaka	S. Mbandaka	CIP,CLR,AMP	*qnrS,blaTEM-1*	
N53	W018	3	Wakiso	S. Newport	CIP,SUL	*qnrS*	
N54	W018	3	Wakiso	S. Bolton	CIP	*qnrS*	
N55	W018	3	Wakiso	S. Bolton	CIP	*qnrS*	
N56	M047	1	Masaka	S. Mbandaka	CIP,CLR,AMP	*qnrS,cmlA,blaTEM*	
N57	W006	1	Wakiso	S. Newport	CIP		
N59	W018	3	Wakiso	S. Bolton	CIP		
N61	W010	1	Wakiso	S. Bolton	CIP		
N64	M063	2	Masaka	S. Heidlberg			
N65	L083	1	Lira	S. Bolton			
N66	M063	2	Masaka	S. Aberdeen			
N67	L025	1	Lira	S. Enteritidis			
N68	M067	2	Masaka	S. Aberdeen			
N69	L027	1	Lira	S. Hadar			
N71	W010	1	Wakiso	S. Enteritidis			
N75	L084	1	Lira	S. Kampala			
N76	M054	2	Masaka	S. Aberdeen			
N78	M050	3	Masaka	S. Aberdeen			
N80	L056	2	Wakiso	S. Enteritidis			
N83	M051	2	Masaka	S. Aberdeen			
N87	L025	1	Lira	S. Enteritidis			
N88	M010	2	Masaka	S. Heidlberg			

N91	L027	1	Lira	S. Hadar			
N92	M037	1	Masaka	S. Kampala			
N94	M052	2	Masaka	S. Aberdeen			
N96	M052	2	Masaka	S. Aberdeen			
N97	L057	1	Lira	S. Aberdeen			

(CIP, ciprofloxacin; SUL, sulphonamide; TET, tetracycline; TRIM, trimethoprim; SXT, sulphamethoxazole/trimethoprim; CLR; chloramphenicol; AMP, ampicillin; ENR; Enrofloxacin) and identified genotypic resistance genes (qnrS, sul1, tetA, dhfrI, dhfrVII, cmlA, blaTEM-1, integrons, dfrA15, aadA1) have been included.
