# Supplementary material for: Diversity and Antimicrobial Resistance Genotypes in Non-Typhoidal Salmonella Isolates from Poultry Farms in Uganda
Source: Int J Environ Res Public Health. 2018 Feb 13;15(2):324. doi: 10.3390/ijerph15020324 (PMC5858393; doi:10.3390/ijerph15020324)
Supplement: Supplementary file 1 [file ijerph-15-00324-s001.zip › ijerph-257722-Figure S2.pdf]

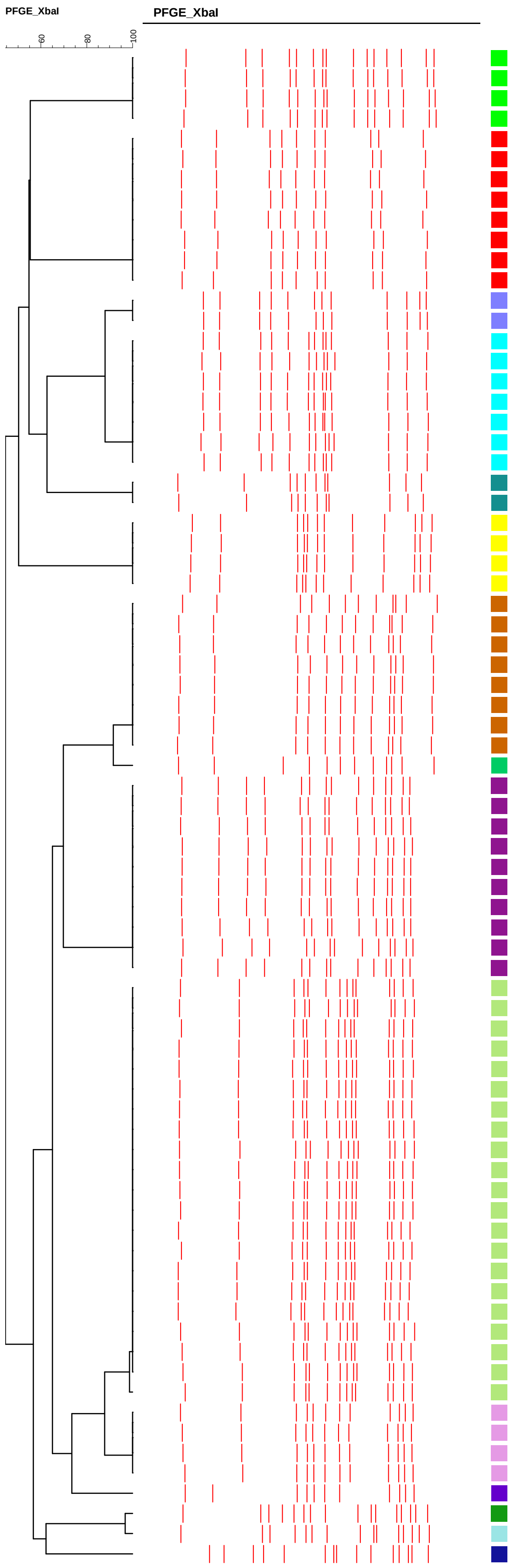


| Farm | Isolate | Serotype | District | Farm size | Pulsotype |
|---|---|---|---|---|---|
| W035 | N45 | Salmonella Mbandaka | 1 | 3 | N |
| W042 | N46 | Salmonella Mbandaka | 1 | 3 | N |
| M047 | N48 | Salmonella Mbandaka | 3 | 1 | N |
| M047 | N56 | Salmonella Mbandaka | 3 | 1 | N |
| L002 | N19 | Salmonella Bolton | 2 | 2 | D |
| L002 | N20 | Salmonella Bolton | 2 | 2 | D |
| L002 | N21 | Salmonella Bolton | 2 | 2 | D |
| W006 | N22 | Salmonella Bolton | 2 | 1 | D |
| W018 | N54 | Salmonella Bolton | 1 | 3 | D |
| W018 | N59 | Salmonella Bolton | 1 | 3 | D |
| W010 | N61 | Salmonella Bolton | 1 | 1 | D |
| L083 | N65 | Salmonella Bolton | 2 | 1 | D |
| M063 | N64 | Salmonella Heidelberg | 3 | 2 | A |
| M010 | N88 | Salmonella Heidelberg | 3 | 2 | A |
| L004 | N35 | Salmonella Heidelberg | 2 | 2 | B |
| L004 | N36 | Salmonella Heidelberg | 2 | 2 | B |
| L050 | N37 | Salmonella Heidelberg | 2 | 1 | B |
| L011 | N38 | Salmonella Heidelberg | 2 | 1 | B |
| L043 | N39 | Salmonella Heidelberg | 2 | 1 | B |
| W035 | N42 | Salmonella Heidelberg | 1 | 3 | B |
| W035 | N43 | Salmonella Heidelberg | 1 | 3 | B |
| L084 | N75 | Salmonella Kampala | 2 | 1 | C |
| M037 | N92 | Salmonella Kampala | 3 | 1 | C |
| L025 | N67 | Salmonella Enteritidis | 2 | 1 | I |
| W010 | N71 | Salmonella Enteritidis | 1 | 1 | I |
| L056 | N80 | Salmonella Enteritidis | 2 | 2 | I |
| L025 | N87 | Salmonella Enteritidis | 2 | 1 | I |
| W003 | N166 | Salmonella Zanzibar | 1 | 1 | E |
| W025 | N181 | Salmonella Zanzibar | 1 | 3 | E |
| M050 | N182 | Salmonella Zanzibar | 3 | 3 | E |
| W003 | N187 | Salmonella Zanzibar | 1 | 1 | E |
| W026 | N193 | Salmonella Zanzibar | 1 | 3 | E |
| W026 | N194 | Salmonella Zanzibar | 1 | 3 | E |
| M050 | N195 | Salmonella Zanzibar | 3 | 3 | E |
| W025 | N196 | Salmonella Zanzibar | 1 | 3 | E |
| M037 | N108 | Salmonella Uganda | 3 | 1 | M |
| W025 | N104 | Salmonella Aberdeen | 1 | 3 | F |
| W026 | N105 | Salmonella Aberdeen | 1 | 3 | F |
| M063 | N66 | Salmonella Aberdeen | 3 | 2 | F |
| M067 | N68 | Salmonella Aberdeen | 3 | 2 | F |
| M054 | N76 | Salmonella Aberdeen | 3 | 2 | F |
| M050 | N78 | Salmonella Aberdeen | 3 | 3 | F |
| M051 | N83 | Salmonella Aberdeen | 3 | 2 | F |
| M052 | N94 | Salmonella Aberdeen | 3 | 2 | F |
| M052 | N96 | Salmonella Aberdeen | 3 | 2 | F |
| L057 | N97 | Salmonella Aberdeen | 2 | 1 | F |
| W059 | N01 | Salmonella Newport | 1 | 3 | H |
| W059 | N02 | Salmonella Newport | 1 | 3 | H |
| W059 | N03 | Salmonella Newport | 1 | 3 | H |
| L042 | N04 | Salmonella Newport | 2 | 1 | H |
| L042 | N05 | Salmonella Newport | 2 | 1 | H |
| L042 | N06 | Salmonella Newport | 2 | 1 | H |
| W015 | N09 | Salmonella Newport | 1 | 3 | H |
| M042 | N10 | Salmonella Newport | 3 | 3 | H |
| M042 | N11 | Salmonella Newport | 3 | 3 | H |
| M053 | N12 | Salmonella Newport | 3 | 2 | H |
| M070 | N16 | Salmonella Newport | 3 | 3 | H |
| M070 | N17 | Salmonella Newport | 3 | 3 | H |
| M070 | N18 | Salmonella Newport | 3 | 3 | H |
| M056 | N26 | Salmonella Newport | 3 | 2 | H |
| M015 | N27 | Salmonella Newport | 3 | 1 | H |
| M015 | N28 | Salmonella Newport | 3 | 1 | H |
| M056 | N29 | Salmonella Newport | 3 | 2 | H |
| M055 | N30 | Salmonella Newport | 3 | 1 | H |
| W042 | N47 | Salmonella Newport | 1 | 3 | H |
| W006 | N57 | Salmonella Newport | 1 | 1 | H |
| W018 | N53 | Salmonella Newport | 1 | 3 | H |
| W015 | N08 | Salmonella Hadar | 1 | 3 | G |
| W017 | N24 | Salmonella Hadar | 1 | 3 | G |
| W017 | N25 | Salmonella Hadar | 1 | 3 | G |
| W015 | N40 | Salmonella Hadar | 1 | 3 | G |
| L027 | N69 | Salmonella Hadar | 2 | 1 | O |
| L067 | N114 | Salmonella Hadar | 2 | 1 | J |
| L067 | N136 | Salmonella Hadar | 2 | 1 | K |
| L021 | N31 | Salmonella Typhimurium | 2 | 1 | L |
