# Supplementary figures and images for: Diversity and Antimicrobial Resistance Genotypes in Non-Typhoidal Salmonella Isolates from Poultry Farms in Uganda
Source: Int J Environ Res Public Health. 2018 Feb 13;15(2):324. doi: 10.3390/ijerph15020324 (PMC5858393; doi:10.3390/ijerph15020324)

Figure S1: A map showing study areas of Wakiso, Lira, and Masaka in Uganda

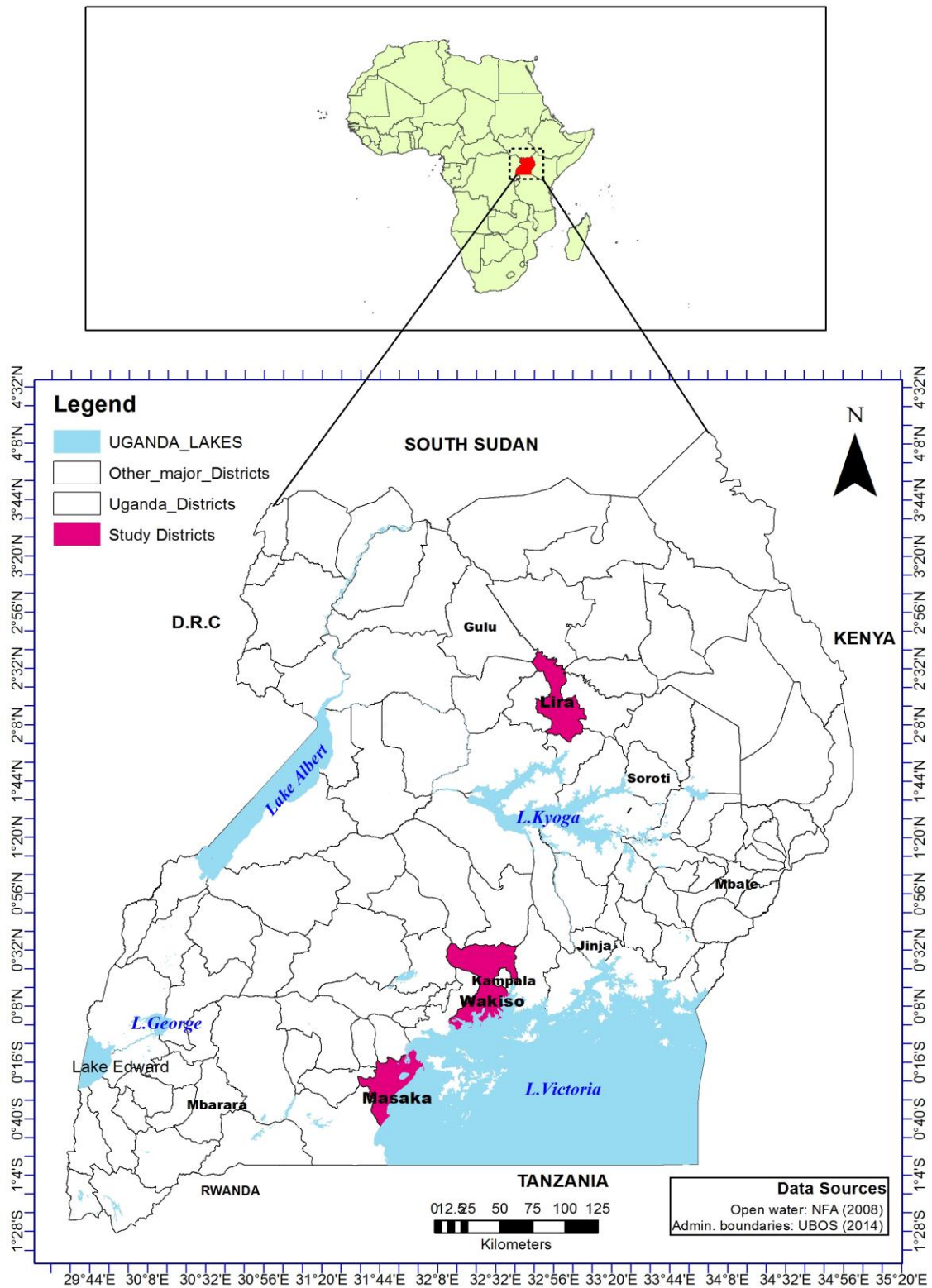

Supplement: Supplementary file 1 [file ijerph-15-00324-s001.zip › ijerph-257722-sup.Figure S1.pdf]
